# Correction: Wang et al. TFRC Ablation Induces Insufficient Cartilage Development Through Mitochondrial p53 Translocation-Mediated Ferroptosis. *Int. J. Mol. Sci.* 2025, *26,* 2724

**DOI:** 10.3390/ijms27073217

**Published:** 2026-04-02

**Authors:** Yidi Wang, Xi Wen, Yutong Guo, Yixiang Wang, Yan Gu

**Affiliations:** 1 Department of Orthodontics, Peking University School and Hospital of Stomatology & National Center for Stomatology & National Clinical Research Center for Oral Diseases & National Engineering Research Center of Oral Biomaterials and Digital Medical Devices & Beijing Key Laboratory of Digital Stomatology & NHC Key Laboratory of Digital Stomatology & NMPA Key Laboratory for Dental Materials, No. 22, Zhongguancun Avenue South, Haidian District, Beijing 100081, China; 2 Central Laboratory, Peking University School and Hospital of Stomatology, No. 22, Zhongguancun Avenue South, Haidian District, Beijing 100081, China

## 1. Figure Legend

In the original publication [1], there was a mistake in the legend for Figure 7. ‘The TFRC-SLC39A14 expression’ in the legend was misspelled as ‘The FRC-SLC39A14 expression’. The correct legend appears below.

**Figure 7.** The TFRC–SLC39A14 expression switch regulates ferroptosis through mitochondrial p53 translocation in ATDC5 cells (**A**) Using the String website (cn.string-db.org) to predict the correlation between protein molecules, it was found that p53 were highly correlated with TFRC, SLC39A14, and ferroptosis-related signaling molecules. (**B**) co-IP analysis of protein interaction between p53, SLC39A14 and TFRC. (**C**) Using Hdock (hdock.phys.hust.edu.cn) to dock ZIP14 and p53 interaction. The result of molecular docking showed that the binding energy of p53 and SLC39A14 was −320.84 kcal/mol. The residues around the protein–protein interaction interface could form hydrogen bonds. The interaction mode of docking results was analyzed by Pymol 2.3.0. The amino acid residue GLY-223, GLU-225, LEU-108 and ASN-103 of p53 formed hydrogen bonds with TYR-154, CYS-158, CYS-165, THR-471 and TYR-187 of SLC39A14, respectively. The length of hydrogen bonds was 2.0 Å, 3.8 Å, 3.4 Å, 2.8 Å and 3.0 Å. These hydrogen bonds could help stabilize protein–protein complexes. (**D**) Western blot analysis of SLC39A14 expression after treatment with proteasome inhibitor MG132. (**E**) Western blot analysis of ubiquitination level of SLC39A14 after *p53* overexpression, *Tfrc* overexpression and *Tfrc* knockdown. (**F**) Western blot analysis of mitochondrial p53 and cytoplasmic p53 level in *siNC* and *siTfrc* groups. (**G**) Fluorescence images of cells and mitochondria to detect p53 translocation in *siNC* and *siTfrc* groups. (**H**) Western blot analysis of mitochondrial p53 and cytoplasmic p53 level in *OE-EV* and *OE-Tfrc* groups. (**I**) Fluorescence images of cells and mitochondria to detect p53 translocation in *OE-EV* and *OE-Tfrc* groups. (**J**) Calcein AM fluorescence probe was used to detect mitochondrial permeability in *siNC* and *siTfrc* groups. (**K**) Calcein AM fluorescence probe was used to detect mitochondrial permeability in *OE-EV* and *OE-Tfrc* groups. (**L**) The images of fluorescence microscope detecting ROS (MitoSOX red) generated by mitochondria in *siNC* and *siTfrc* groups. (**M**) The images of fluorescence microscope detecting ROS (MitoSOX red) generated by mitochondria in *OE-EV* and *OE-Tfrc* groups.

## 2. Error in Figure

In the original publication, there was a mistake in Appendix B Figure A2 as published. It was identified that the WB image for ACAN in Appendix B Figure A2B was an unintentionally duplicated image. The corrected whole Figure A2 appears below.

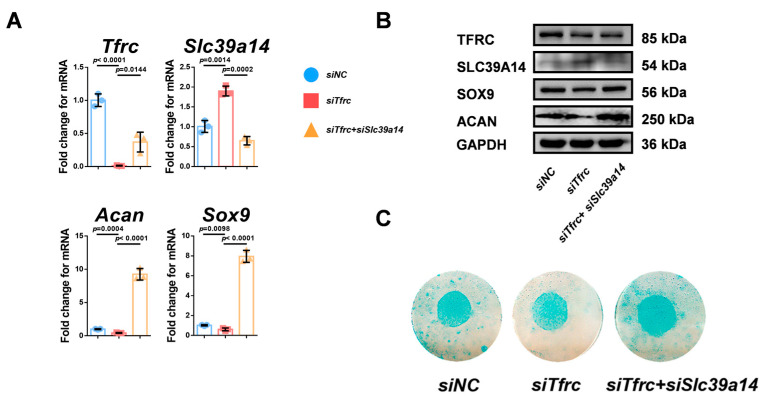



The authors state that the scientific conclusions are unaffected. This correction was approved by the Academic Editor. The original publication has also been updated.

## References

[1] Wang Y., Wen X., Guo Y., Wang Y., Gu Y. (2025). TFRC Ablation Induces Insufficient Cartilage Development Through Mitochondrial p53 Translocation-Mediated Ferroptosis. Int. J. Mol. Sci..

